# Round the Hospitals

**Published:** 1921-02-26

**Authors:** 


### ROUND THE HOSPITALS.

For some time information has been anxiously awaited concerning the proposed Prisons Nursing Staff, and the necessary provision of a sufficient trained staff to carry on the work of nursing in the hospitals at Holloway and elsewhere. Many months ago the appointment of Miss L. Jolley to be matron-in-chief of this Service was announced, and it was hoped that this would be followed by the organisation of adequate arrangements for a trained nursing staff, with a definite status, separate accommodation, and suitable conditions of employment. No official information has yet been forthcoming, but we understood that a Nursing Board has been formed by the Prisons Commission at the Home Office for the prison hospitals in England and Wales, and that on this Board are Dr. Mary Scharlieb, Dame Sarah Swift, Miss McIntosh (Matron of St. Bartholomew's Hospital), and Miss Hogg (Matron of Guy's Hospital). This is a move in the right direction. We hope that every encouragement will be given to those who have joined the Board and that the recommendations they may put forward will be promptly considered and acted upon. The ladies we have mentioned are experts not only in nursing matters but in conditions affecting the well-being of the race, and we await with confidence an early announcement which shall assure the public that the nursing needs of sick prisoners and the just requirements of the nurses in attendance upon them will henceforth be adequately met.

---

The new number of the College *Bulletin* grapples boldly with nomination and election problems, and members should carefully study the figures of attendances at meetings of those they elect to the Council. The intimate tone adopted towards members is specially valuable. By far the greater majority never come into any kind of contact with headquarters save through the medium of this quarterly communication, and it is a mark of high editorial skill so to address the readers that they are brought into the centre of the difficulties, hopes, and fears experienced by the permanent officials. By this means the *Bulletin* does much to strengthen the slight tie created by the act of joining the College. Whether the link ultimately becomes vigorous enough to influence the life and outlook must, of course, depend on the individual member. The effect of bare and curt officialism is to snap it once and for ever. But the note of confidence and comradeship which inspires the *Bulletin* is favourable to the growth of strong corporate fellowship.

---

For how many years has there not been an intermittent agitation for a registered nurses' uniform protected by law with a registered nurses' badge? Readers will note that both are now practically within their grasp. The General Nursing Council under the powers committed to it by the Nurses' Registration Act is taking steps with a view to designing a protected uniform and badge which shall be acceptable to the great body of nurses. Do nurses desire a cloak and bonnet or a coat and hat? Strong opinions are held in favour of each, and nurses are invited to express their individual choice by writing to the Registrar. (See instructions, page vi.) The response to this request is likely to afford a good test of the number of "live" members in the profession. The College of Nursing recently received 3,000 replies to the questions addressed direct to their members on the question of Unemployment Insurance and Hours of Labour.

**ROUND THE HOSPITALS**—(*continued*).

Allowing about 4,000 on account of deaths, marriage, retirement, emigration and other causes which detach members from professional interests, this total represents about a quarter of the remaining number. We shall be very much surprised if the number of replies received by the Registrar on the present occasion exceeds this total. But it is a great opportunity.

Conflicting reports reach us of the death of Mademoiselle Thulliez, who was one of Miss Edith Cavell's devoted colleagues at Brussels. Rumour has it that recent documents have revealed her complicity with those who denounced the architect, Philippe Baucq, to the Germans, and that she has committed suicide to escape from justice. The facts as we know them certainly do not bear out this sinister imputation on Louise Thulliez's honour. She was decorated in 1919 by M. Clemenceau with the Cross of the Legion of Honour and the Croix de Guerre with palms, and was publicly acclaimed for "a devotion comparable with that of Miss Edith Cavell." More conclusive—and, indeed, quite unanswerable—is the fact that she was twice sentenced to death by German courts-martial for helping Entente soldiers to escape, and was only rescued from paying the extreme penalty by the intervention of the Spanish Embassy, through which the sentence was commuted to one of penal servitude. She served nearly three years of hard labour, and if she has now taken her own life, of which there is at present no certain evidence, it is reasonable to conclude that it was the hardships she then endured which have mentally affected her.

We are glad to learn that the Committee of the Queen's Hospital for Children has sanctioned a higher scale of salaries for its nursing staff, and that the increased salaries come into force as from February 1. Under the new scale the assistant matron will receive £100 instead of £90, rising by annual increments of £10 to £140; the home sister £85 instead of £75, rising by £5 annually to £125; the night sister, whose salary has hitherto been £70, is in future to be paid the same as the home sister; ward sisters will receive £80, rising £5 annually to £120, instead of £60 to £70; and staff nurses £50, rising £5 annually to £60. The new scale is substantially the same as that recommended by the representatives of the principal London Children's Hospitals meeting in conference, though it varies in some respects from that recommended by the College of Nursing.

Military nurses, which term includes, besides the Q.A.I.M.N.S. and its Reserve, nurses of the Territorial Force Nursing Service, assistant nurses, nurses in military families' hospitals, V.A.D. nursing members and special probationers when serving in military hospitals and in receipt of pay direct from Army funds, are after March 1 to be granted the privilege when proceeding on furlough of making the double railway journey at a single fare, providing the furlough is for not less than seven days. The procedure to be followed is for nurses to pay their own fares at the ordinary current single rate from their station (or port of disembarkation in the case of those coming from abroad) to their leave address, and for the return journey therefrom to their station or port of embarkation each nurse will receive a railway warrant.

Heartiest congratulations are rendered to Miss Annie Louise McIlroy, O.B.E., M.D., Ch.B., D.Sc. (Glasgow), on her appointment at a stipend said to be £2,000 a year to the University Chair of Obstetrics and Gynæcology tenable at the London School of Medicine for Women. Miss McIlroy studied at the Universities of Glasgow, London, Berlin, Vienna, and Paris, and holds the diploma in midwifery of the Rotunda Hospital, Dublin. For four years she rendered admirable war service as Surgeon-in-Charge of the Girton and Newnham Unit of the Scottish Women's Hospitals for Foreign Service, stationed at Salonika and Belgrade, and has since been gynæcological specialist in the 82nd General Hospital at Constantinople. As Professor she will be Director of the Obstetrical and Gynæcological Unit at the Royal Free Hospital. In her appointment all women are conscious of a glow of achievement. It is a historic example of the tendency shown in the working out of all great ideals to surpass the original conception. It was Florence Nightingale who first dreamed of the woman gynæcologist, and she lived long enough to watch the remarkable fulfilment of her vision.

The following nurses are on the eve of departure for the Mission field, or have already started: Miss Biggs, member of the Australian C.M.S., for Toro; Miss Lamb and Miss Hopkins for Old Cairo; Miss Cornwall for Palestine; Miss Birch for Persia; Miss Weatherell and Miss Snowdon for Hangchow. Mrs. Junkison has been appointed dispenser to the Persia Mission. The Church Missionary Society publishes statistics of the medical and nursing staffs in various hospitals under their ægis, and we are not at all satisfied that medical mission work can be properly carried on, with due regard to the well-being of the nurses, under the conditions imposed. For instance, at the Pakhoi Hospital, in South China, with 210 beds, we find two nurses and no doctors. Ought two nursing sisters to be left alone, with only such native help as they can get, in a hospital of this size? We fear neither Christianity nor medical science can reap much glory from hospital work thus attempted. Another bad example is Peshawur, where one doctor and two nurses cope with 100 beds, and the out-patients total 20,743 in a year. The total number of C.M.S. hospitals is thirty-seven, containing 3,577 beds in all, with a total of nearly 40,000 in-patients and 853,632 out-patients in the year. It is deplorable to learn that but seventy-five doctors and seventy-nine nurses, with seven auxiliary workers, are being set to a task of this magnitude.

**ROUND THE HOSPITALS**—(*continued*).

Registration for midwives is a new thing in Scotland. Before the Board was set up five years ago midwifery did not attract the class of women likely to do it credit, and Sir W. Leslie Mackenzie, presiding at the annual meeting of the Scottish Midwives Association, held in the Nurses' Club, Edinburgh, declared that in one large Scottish city with a population of 400,000 the practising midwives numbered less than a dozen. He believed the effect of registration would be steadily to improve the midwife's professional education and to increase the confidence of the community in her special services.

Packed from floor to ceiling the London Coliseum was a wonderful sight on Tuesday, February 22, when a matinee was given to help the National Hospital for the Paralysed and Epileptic in its serious financial difficulties. And another purpose of the matinee, on the part of Sir Oswald Stoll, the Coliseum's proprietor, was to recognise the ungrudging and voluntary work given by Dr. T. Grainger Stewart of the National Hospital in visiting the disabled warriors at the War Seal Foundation. We hope the financial success of the matinee (£2,000 we understand has been received up to the present), both direct and indirect, may prove to be as great as was the generosity of the artistes and the organising ability displayed. The particularly gay and delightful second act of "The Beggar's Opera," with the company and orchestra from the Lyric Theatre, Hammersmith, the pathetic and charming "Pantaloon," by Sir James Barrie, with such incomparable artists as Karsavina, Novikoff, and Albert Chevalier; the comedietta by Lady Hugh Bell, in which Mr. Charles Hawtrey was his own inimitable self; and last, but by no means least, M. Mark Hambourg's beautiful playing of the Wedding March by Mendelssohn-Liszt, to which he generously added an encore, and the hilarious and completely delightful medical "Rag" by students of the St. Thomas's, St. Bartholomew's, and the London Hospitals, provided a gamut for every emotion and kept the huge audience interested, amused, and pleased from first to last. Her Majesty the Queen, with H.R.H. Princess Mary, was present during the whole afternoon.

It appears that there is a great need for a proper training course for health visitors in the North of England. There is at present no institution training health visitors between Edinburgh and Leeds. The country ought to be in a position to train its own staff, and it is hoped that the long-promised course in connection with Armstrong College and the College of Medicine will soon come into operation. The duties required of health visitors render it essential that they should be thoroughly grounded in social science. Regarding the Board of Education diploma, the superintendent says it will be necessary for the county regulations to be altered to recognise this diploma as qualifying holders for appointment in the county. In cases where the candidate is not also a certified midwife or a registered nurse, if appointed, she should not be entitled to any increase of salary until she obtains the C.M.B. Certificate, which she should ordinarily do within two years of her appointment, "study leave" being granted her for the purpose on the usual terms. The superintendent suggests that the age-limit be reduced from twenty-five to twenty-three years.

As a memorial to the work of medical women in Serbia during the war a fund to provide medical scholarships for Serbian girls is to be established, and was initiated at a recent meeting at the house of Lady Swaythling. It is hoped to collect sufficient money to establish two scholarships, the estimated amount required for each being £4,500. They will provide maintenance and education for a medical degree at the London School of Medicine for Women and the Royal Free Hospital, and will be open to girls from any part of Jugo-Slavia, who will be required to undertake to return to practice in their own country. We can conceive no simpler way of cementing the affection which grew up during the war between the people of Serbia and of this country, nor of giving to this island people a knowledge of the ideals, standards, and qualities of an alien race. Dr. Mary Scharlieb is the President of the fund, and donations may be sent to Mrs. H. Flinders Petrie, at 21 Wimpole Street, W. 1.

We learn that the holiday home for nurses for which £10,000 has been given by the United Services Fund is to be for trained nurses and V.A.D. members who served in the war. The United Nursing Services Club is to be for trained nurses only, and this Club, according to present arrangements, will not have the management of the holiday home.

Lady Helena Gibbs, in co-operation with the Waifs and Strays Society, has presented Bristol with a beautiful little Babies' Home, to be called the Victoria Gibbs Memorial Home for Babies. Twenty-three babies are already in residence under Miss Clive Rounds as matron, and those who have no homes will be passed on to the Waifs and Strays Orphanage as they grow older.

We commend the action of (to quote the headline in the local paper) "the nurse who wouldn't stay." She took service last November under the Risbridge Board of Guardians in East Anglia, and resigned in January. She stated her reasons as follows: The situation of the institution so far from a town or railway station, with the expense attached to taking reasonable recreation; the insufficiency of the ration allowance, reducing the value of her salary; the lack of any person to attend to the nurses' rooms; the defective arrangements of bathing and lavatory conveniences and of hot water supply at night; no whole day's leave once a month. This is a summary of defects which are all, except the first, remediable and necessary to be remedied. We fear Risbridge is but one among many rural institutions where such defects exist without any attempt being made to find the remedies.

